# Successfully treated unusual case of primary adrenal and spinal tuberculosis with three years follow up

**DOI:** 10.11604/pamj.2014.17.108.2575

**Published:** 2014-02-13

**Authors:** Biswas Shrestha, Ahmed Omran, Pengfei Rong, Wei Wang

**Affiliations:** 1 Department of Imaging and Interventional Radiology, The Third Xiangya Hospital of Central South University, Changsha, Hunan, China; 2 Department of Pediatrics and Neonatology, Suez Canal University, Ismailia, Egypt

**Keywords:** Adrenal insufficiency, adrenal tuberculosis, follow up, spinal tuberculosis

## Abstract

The global increase in incidence of Tuberculosis (TB) is returning to be a major health issue grabbing a universal concern. Although extrapulmonary tuberculosis (EPTB) has a broad spectrum of clinical manifestations, primary involvement of the adrenal glands along with spine without pulmonary affection has been rarely reported. We report a case of successfully treated adult Asian male patient presented with primary adrenal TB, complicated with chronic adrenal insufficiency accompanied with upper lumber spinal TB. We also present the follow up of our patient after three years.

## Introduction

Tuberculosis (TB) remains a major public health problem, ranking as the second leading cause of death from an infectious disease worldwide, after the human immunodeficiency virus (HIV). World Health Organization (WHO) reported more than 2 billion people, equal to one-third of the world's population, are infected with TB bacilli and 1.3 billion people die from TB. In 2011 alone, WHO reported more than 9 million new cases of TB and 1.4 million TB deaths worldwide [1, 2]. Extrapulmonary tuberculosis (EPTB) is increasing and accounts for one in five of the new diagnosed TB patients [3]. In many part of the world, TB remains one of the leading causes of adrenal insufficiency and it is vital to identify the infectious cause for Addison disease to prevent patient from developing life-threatening adrenal crisis [4]. Adrenal TB is characterized by a peculiar clinical and radiological pattern, and its diagnosis is difficult to obtain even after biopsy [5]. Approximately 50% of skeletal TB involves the spine, most commonly affecting the lower thoracic and upper lumbar levels [6–8]. We here report a successfully treated unusual case of primary combined adrenal and spinal TB complicated with chronic adrenal insufficiency, with a combination of anti-TB drugs and steroid therapy. We also present a three years follow up for our patient showing significant changes in the adrenal glands radiological findings.

## Patient and observation

**Figure 1:**
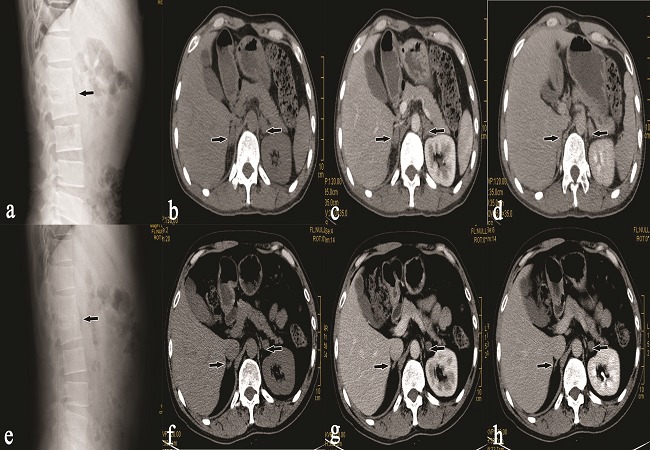
Pre-treatment (a) and post treatment (e) lateral lumbar plain film showing fusion and irregular osteolytic destruction of the L1, L2 vertebral bodies (arrow). Pre-treatment (b, c, d) and Post-treatment (f, g, h) CT- abdomen of different phase showing bilateral enlargement of adrenal gland (upper arrow) with irregular enhancement and lesion like changes and decrease in adrenal gland size (lower arrow) and significant reduction in adrenal lesions respectively

A 36 year old male from Asian origin presented on 13/10/2009 with hyperpigmentation and darkening of the skin that had been developed for previous 5 months accompanied by fatigue for a period of more than a year with lumbar back pain. During his physical examination, he had a core body temperature of 36°C , a pulse 84 beats per minute, a respiratory rate 20 breaths per minute and a blood pressure of 100/60 mmHg. He had mild pallor, but no jaundice or cyanosis. Skin showed generalized hyperpigmentation with prominent gingival and buccal mucosa darkening. There was no significant peripheral lymphadenopathy. Thyroid gland was not enlarged. His abdomen was soft and flat with no organomegaly. Chest and cardiac examinations revealed no abnormalities. Laboratory findings included a red blood cell count of 4.51 × 10^12^/ L; hemoglobin 11.1 g/dl; white blood cell count 16.31 × 10^9^/ L; (neutrophils 61.6%; lymphocytes 13.1%; monocytes, 7.0%; eosinophils, 0.1%; basophils, 0.7%); platelet count 308 × 10^9^/ L; high erythrocyte sedimentation rate (42 mm/h), high C-reactive protein (14.88mg/L), low-serum sodium (129 mmol/L), high-serum potassium (5.4 mmol/L), low serum chloride (92 mmol/L), blood glucose level (118 mg/dl), normal kidney function, normal liver function, low-serum cortisol 8 am (2.9 μg/dL), 4 pm (2.6 μg/dL), TB IgM antibody (-ve), TB IgG antibody (+ve) and positive purified protein derivative test (PPD). A Chest x-ray revealed no abnormalities. A Thoracic lumbar plain film revealed narrowing of the L1- L2 space with irregularity and osteolysis of the endplates suggesting the possibility of lumbar spine TB (Figure 1 - A). An Abdominal CT imaging revealed bilateral adrenal mass like enlargement with irregular enhancement and lesion like changes without calcification (Figure 1 - b-d). Based on the clinical presentation, laboratory findings of positive IgG anti-TB antibodies, positive PPD test, High ESR, low serum cortisol, low sodium level and high potassium level, beside the radiological findings of spinal and adrenal involvement, the diagnosis of combined primary adrenal and spinal TB with adrenal insufficiency was highly suggested.

Based on this diagnosis, the patient started anti-TB drugs in the form of (isoniazid, rifampicin, ethambutol, pyrazinamide) in combination with vitamin B6 tablets and steroid therapy in the form of prednisolone (7.5 mg/day) in two divided dose of 5 mg in the morning 8 am and 2.5 mg afternoon at 4 pm. He was called for clinical follow up every 6 months and all the necessary investigations were performed each time.

In the recent follow up of our patient in 2012, he was without clinical symptoms and all the laboratory investigations were nearly normalized. The anti TB drugs were stopped but steroid therapy in the form of prednisolone is continued with same dose as earlier. The thoracic lumbar plain film taken later in 2012, that we have presented in the article demonstrated nearly the same finding as shown in the earlier film taken during the initial presentation (Figure 1-e). A CT scan of his adrenal glands documented a relevant reduction of the bilateral adrenal masses and the adrenal lesions were significantly reduced than the earlier CT film along with diminished degree of enhancement (Figure 1-f-h), however it is not conclusive about the restoration of its normal adrenal function.

## Discussion

TB is one of the most ancient multi-systemic, granulomatous infectious diseases recorded in human history. However, its unusual presentations still elude physicians even in this era of advanced medicine. Although EPTB has a broad spectrum of clinical manifestations, primary involvement of the adrenal gland and spine with long term follow up has been very rarely reported. We herein report a rare occurrence of adrenal insufficiency secondary to primary adrenal TB accompanied by spinal involvement and its long term follow up after 3 years from onset of the diagnosis.

The patient had EPTB, both adrenal and spinal, resulting in adrenal insufficiency and lumbar back pain. For several months prior to the presentation, back pain had been present and this may be evidence that hematogenous spread to the adrenal glands occurred from the lumbar vertebrae.

Although adrenal TB is the major cause of chronic primary adrenal insufficiency, especially in developing countries, common findings of Addison's disease does not usually appear until more than 90% of adrenal tissue has been destroyed [4], explaining the chronic nonspecific nature of the symptomatology in our patient and positive anti-TB IgG which indicate the chronicity of infection.

In our patient, the adrenal involvement was in the form of bilateral mass enlargement; Yang et al reported that bilateral involvement can be used as one of the most important discriminators of TB from a primary tumor in the adrenal gland [9].

Adrenal TB usually occurs together with the presence of extra-adrenal TB and in our case it is associated with lumbar spine involvement. In skeletal TB, the upper lumbar spine is most commonly affected as seen in our case. The loose internal structure of the disk allows the infection to disseminate more widely into additional spinal segments, resulting in the classic pattern of involvement of more than one vertebral body together with the intervening disks, which exactly occurs in our case [10].

Although adrenal cortex has considerable capacity of regeneration, Addison's disease due to tuberculosis is generally regarded as irreversible. Only a few patients with tuberculosis showed recovery of adrenal function [11]. Very recently Anaforoğlu et al reported that stoppage of steroid therapy for patient with adrenal insufficiency secondary to TB is associated with relapse of the symptoms as seen in our patient and in their 1 year follow up of the adrenal gland by MRI they didn't find any significant radiological changes [12]. However, the improved radiological findings in comparison with the old CT film in our patient is not associated with restoration of the normal function of the adrenal glands; explaining the extension of steroid therapy in our patient.

## Conclusion

Physicians need to be aware of the widely different manifestations of EPTB which is a potential cause of adrenal insufficiency. Even with the improvement on the adrenal glands radiological findings, steroid therapy may be needed to be continued for a long time.
